# Comprehensive DNA methylation profiling by MeDIP-NGS identifies potential genes and pathways for epithelial ovarian cancer

**DOI:** 10.1186/s13048-024-01395-3

**Published:** 2024-04-16

**Authors:** Priyanka Gautam, Sameer Gupta, Manisha Sachan

**Affiliations:** 1 Department of Biotechnology, Motilal Nehru National Institute of Technology, Allahabad, Prayagraj, 211004 India; 2 Department of Surgical Oncology, King George Medical University, Lucknow, India

**Keywords:** Ovarian cancer, MeDIP-seq, Bioinformatics analysis, Differentially methylated regions (DMRs), DNA methylation, QRT-PCR, Biomarker

## Abstract

Ovarian cancer, among all gynecologic malignancies, exhibits the highest incidence and mortality rate, primarily because it is often presents with non-specific or no symptoms during its early stages. For the advancement of Ovarian Cancer Diagnosis, it is crucial to identify the potential molecular signatures that could significantly differentiate between healthy and ovarian cancerous tissues and can be used further as a diagnostic biomarker for detecting ovarian cancer. In this study, we investigated the genome-wide methylation patterns in ovarian cancer patients using Methylated DNA Immunoprecipitation (MeDIP-Seq) followed by NGS. Identified differentially methylated regions (DMRs) were further validated by targeted bisulfite sequencing for CpG site-specific methylation profiles. Furthermore, expression validation of six genes by Quantitative Reverse Transcriptase-PCR was also performed. Out of total 120 differentially methylated genes (DMGs), 68 genes were hypermethylated, and 52 were hypomethylated in their promoter region. After analysis, we identified the top 6 hub genes, namely POLR3B, PLXND1, GIGYF2, STK4, BMP2 and CRKL. Interestingly we observed Non-CpG site methylation in the case of POLR3B and CRKL which was statistically significant in discriminating ovarian cancer samples from normal controls. The most significant pathways identified were focal adhesion, the MAPK signaling pathway, and the Ras signaling pathway. Expression analysis of hypermethylated genes was correlated with the downregulation of the genes. POLR3B and GIGYF2 turned out to be the novel genes associated with the carcinogenesis of EOC. Our study demonstrated that methylation profiling through MeDIP-sequencing has effectively identified six potential hub genes and pathways that might exacerbate our understanding of underlying molecular mechanisms of ovarian carcinogenesis.

**Supplementary Information:**

The online version contains supplementary material available at 10.1186/s13048-024-01395-3.

## Introduction

Ovarian cancer is the fifth most lethal gynecologic malignancy in women globally, mainly affecting women aged 55 to 74 years [1, 2]. It has varied heterogeneity on the molecular, histopathological, and clinical levels, and is linked with the highest fatality rates [3]. According to the American Cancer Society, a total of 19,710 new cases and 13,270 deaths have been recorded in 2023 [4]. There is a strong correlation between survival rate and stage of epithelial ovarian cancer. Early detection of ovarian cancer results in an elevated 5-year survival probability of up to 93%. However, due to the asymptomatic nature of the disease at the early stage (I/II), high recurrence rate, and lack of improved early diagnosis methods, the disease is diagnosed at an advanced stage (stage III/IV) leading to a 5-year survival rate lesser than 35% [5].

At present, the current strategies for ovarian cancer detection involve pelvic examination, transvaginal ultrasonography, and imagining techniques like MRI and PET scans. The drawback of these methods is their limited sensitivity and specificity therefore, they are combined with other serum biomarkers like CA125 and HE4 [6]. Clinically, CA125 is an FDA-approved serum biomarker routinely used for monitoring treatment and disease recurrence with a sensitivity of 50–55% and specificity of 90% [7]. Nevertheless, this sensitivity and specificity are not efficient for early-stage diagnosis and moreover CA125 is found to be elevated in benign conditions and in other non-ovarian malignancies during pregnancy. Due to the lack of early detection methods and less specificity of imaging techniques, there is an urgent need to identify a set of more valuable and reliable molecular markers and to study their role in molecular mechanisms implicated in the development and progression of ovarian cancer, which could further aid in the diagnosis of ovarian cancer.

DNA methylation is one of the most common and well-studied epigenetic modifications. Hypermethylation at the tumor suppressor gene promoters plays a key role in the onset and progression of cancer. Its high stability and occurrence in the early stage of tumorigenesis make it a promising biomarker for early detection [8]. The first direct involvement of altered DNA methylation patterns in carcinogenesis was established in 1994 by Herman et al., in cases of renal carcinoma demonstrating promoter hypermethylation as a factor responsible for the silencing of tumor suppressor gene VHL [9]. Following that, other similar investigations were undertaken, with abnormal methylation at CpG islands in the promoter region as a probable mechanism in the transcriptional suppression of tumor suppressor genes such as RASSF1a, BRCA1, CDH1, DAPK, and OPCML in a variety of cancers [10–12].

Similarly, Barekati et al. explored the aberrant methylation pattern of BMP6, BRCA1, and P16 and suggested their use as biomarkers in breast cancer detection [13]. Promoter hypermethylation as a silencing factor of GSTM2 and PENK in prostate cancer and CFTR gene in breast cancer has been considered with high diagnostic relevance [14, 15].

MeDIP, coupled with massively parallel NGS, is a cost-effective method even suitable for minute clinical samples and has been demonstrated as an effective technique for methylation investigation [16, 17]. Many cancer studies employed this technique to identify DMGs (Differentially Methylated Genes), such as breast cancer [18], ovarian cancer [19], pancreatic cancer [20], and others. In this study, we have analyzed the methylation profile of ovarian cancer samples using methylated DNA immunoprecipitation combined with high-throughput sequencing (MeDIP-seq) by Illumina NextSeq 500 platform, which employs an anti-cytosine antibody resulting in the enrichment of methylated DNA. After sequencing, differentially methylated regions were identified and further validated by targeted bisulfite sequencing to narrow down the CpG site-specific methylation. We have also investigated the expression profiles of selected hypermethylated genes using expression GEO datasets and validated them using Quantitative RT-PCR. Using bioinformatic analysis, we have identified hub genes and pathways that might be involved in ovarian carcinogenesis. Furthermore, a deeper knowledge of the molecular pathways involved in the progression and development of ovarian cancer is required.

## Materials & method

### Sample collection

**Fig. 1:**
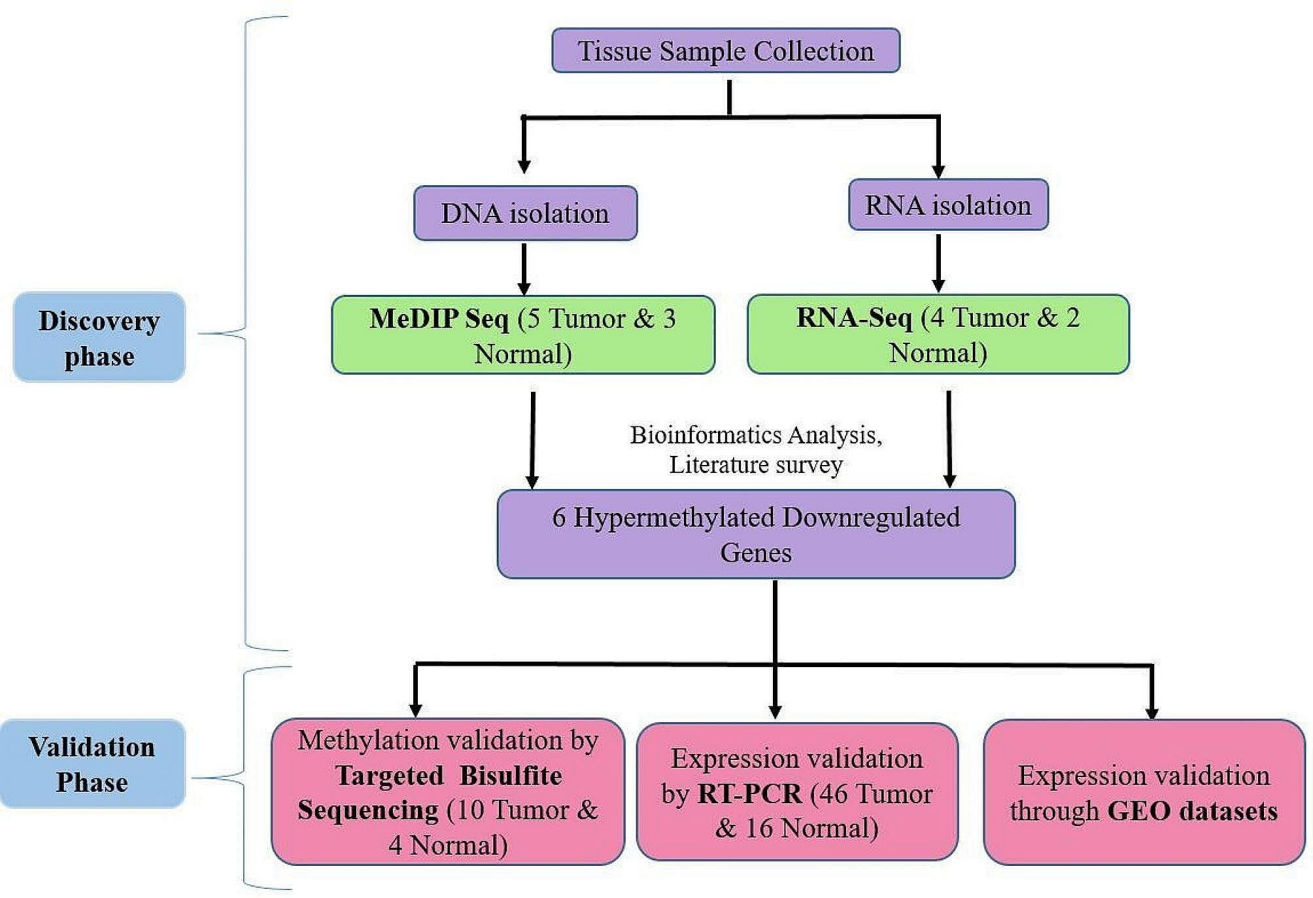
A workflow demonstrating the study design

The ovarian cancer patient samples were collected from King George Medical College, Lucknow, and stored at -80 °C until analysis. We used 65 epithelial ovarian tumor samples and 25 normal control samples obtained from healthy individuals. The majority of tumor samples were serous adenocarcinoma. The clinical information of all the tissue samples is shown in Supplementary Table 1. The study was approved by the Institutional Ethics Committee and informed consent was collected from participants before the study. (Ref. No. IEC/2021-22/05). The workflow of this study is shown in Fig. 1.

### DNA isolation from tissue samples

Genomic DNA was extracted from the frozen ovarian tissue samples using the standard protocol (proteinase K & phenol-chloroform extraction method). After homogenizing the tissue samples (10–30 mg) in 2 ml of SET buffer (5 mM EDTA, 0.3 M sucrose, and 25 mM tris), the samples were centrifuged for 10 min at 6000 rpm. After removing the supernatant, the pellet was mixed with 1 ml of lysis buffer (50 mM Tris, 10% SDS, 2.5 mM EDTA, and 100 mM NaCl) followed by Proteinase K digestion (50 µg/mL) for 12–16 h at 37 °C. The lysate was subjected to phenol: chloroform: isoamyl alcohol (25:24:1) at 25 °C. Subsequently, 1/30 volume of 3 M sodium acetate (pH 5.0) and two volumes of cooled absolute ethanol were used to precipitate the DNA, followed by a 70% ethanol wash. Ultimately, 100–200 µl of TE (10 mM Tris–HCl pH 8 and 1 mM EDTA) was used to re-suspend the DNA pellet, which was then kept at 4 °C for further use. DNA quality and concentration was further determined by agarose gel electrophoresis and spectrophotometry respectively.

### Methylated DNA immunoprecipitation & sequencing

Covaris M220 was used to fragment isolated genomic DNA, producing a mean fragment distribution of 150 bp. End repair was performed on the generated fragments using the End Repair mix, followed by A-tailing and adapter ligation. The samples were then subjected to immunoprecipitation using antibodies against 5mC as per the manufacturer’s instructions (MagMeDIP kit). The methylated DNA was then enriched by a limited number of PCR cycles followed by AMPure XP beads purification. Quality check of the PCR enriched libraries was done on Agilent 4200 Bioanalyzer. After obtaining the Qubit concentration for the library and the mean peak size from the Bioanalyzer profile, PE Illumina libraries were then loaded onto the Illumina NextSeq 500 platform (Illumina, CA, USA) for sequencing.

### Bioinformatic analysis

#### Data preprocessing and identification of differentially methylated regions

The Trimmomatic [v0.35] programme was used to remove adaptor sequences, ambiguous reads, and low-quality sequences from the received MeDIP-Seq raw data to produce high-quality reads. High-quality (QV > 20) paired-end reads were mapped to the human reference Hg19 genome (GRCh37) using the BWA-Mem software. Further alignment files were analyzed by the samtools (V1.6) to convert the alignment output into the BAM file. Only those files with Properly Paired Read Pair Tag and Mapping Quality of 1 and above were retained, and the rest were excluded from the analysis. Direct screening was performed on Bam files to look for methylated areas. To analyze methylated genome regions, diffReps (v 1.55.6) was used with a sliding window of 1 kb, and reads falling into this region were counted. Any overlapping regions obtained were merged and re-evaluated. Additionally, diffReps computes the *p*-value and q-Values by performing the G-Test on the log fold change values and interpret the hyper/hypomethylation based on the normalized read count. For the annotation of methylated genomic fragments, tool region analysis (v 1.0) was used, and all the information about genes were taken from Ensemble while performing annotation. The *p*-value < 0.05 and |log2FC| > 0.2 for hypermethylated and |log2FC| < 0.2 for hypomethylated genes were set as the cut-off criterion for further analysis.

#### Functional and pathway enrichment analysis

An online biological information database known as the Database for Annotation, Visualisation, and Integrated Discovery (DAVID, version 6.8; http://david.ncifcrf.gov) was utilized to examine the gene ontology of DMGs [21]. This offers functional analysis according to three categories: Kyoto Encyclopaedia of Genes and Genomes (KEGG) pathway analysis, Cellular Components (CC), Molecular Functions (MF), and Biological Processes (BP). *P*-value < 0.05 was used as the cut-off criterion for statistical significance.

#### PPI network construction

Search Tool for the Retrieval of Interacting Genes (STRING) (http://string-db.org/; version 11.0) is an online database for identifying the interaction between different proteins based on information fetched from sources like text mining, experiments, databases, and predictive bioinformatics data [22]. To find out the relationship between DMGs, we have constructed a network using the STRING database and set a minimum interaction score > 0.40, which indicates the medium confidence between the interactions of two proteins [23]. Cytoscape (https://cytoscape.org/; version 3.10.1) was then used to visualize the resulting network from STRING [24].

#### Validation with targeted bisulfite sequencing

Next, we validated the consistency of methylation level of 6 gene promoters through targeted bisulfite sequencing on the Illumina Nextseq 500 platform. For this, we included DNA of ten tumor and four normal control samples. First, the EZ DNA METHYLATION-GOLD kit was used for bisulfite conversion of all DNA samples. (#D5005, zymo). After conversion, primers were designed using Bisulfite Primer Seeker with provided contigs followed by amplification of the bisulfite converted DNA by conducting the Bisulfite specific PCR. Amplified fragments were checked on 2% agarose gel. Finally, libraries were constructed using the NEBNext Ultra II FS DNA Library Prep Kit for Illumina. Primer details are shown in Table 1. The library preparation involves the addition of 100ng amplicon to NEBNext Ultra II FS Enzyme Mix at 37 °C for 5 min to get the fragment size of 200-450 bp. After fragmentation, adapter ligation was done by mixing NEBNext Ultra II Ligation Master Mix and NEBNext Adaptor for Illumina with fragmented DNA and enzyme at 37 °C for 15 min. Next, the cleanup of adapter-ligated DNA was performed, followed by PCR enrichment of adaptor-ligated DNA. After enrichment, library quality was accessed on a D1000 screen tape station, and concentration was checked by Qubit, followed by sequencing. These libraries were sequenced in a 2*150 bp paired-end run using the NovaSeq 6000 with v1.5 reagents (300 cycles). After sequencing, raw data was generated and processed for downstream analysis. Reads were filtered using the Fastx toolkit and Fastp. Alignment to the reference genome to methylation calling and coverage estimation is performed using a Bismark tool. Further, Methykit was used for the identification of methylated cytosines of the target genes. Homer was used for the annotation and visualization of DMRs, followed by the calculation of the beta value and the mean methylation percentage.

**Table 1:** Sequence information of differentially methylated gene primers for targeted sequencing

**STK4: Forward primer**	**AGTAGAGACGGGGTTTTATC**
Reverse primer	CTAATACCCACCACCTAAAA
**BMP2**: Forward primer	TATGTTGTGGAGGTTTTTTTG
Reverse primer	TCTATCCCAAATCACAAAACT
**CRKL**: Forward primer	TGAAAAGGGAGTGAGTTAGTAG
Reverse primer	TACCTCAACCTCCCAAAATAC
**PLXND1**: Forward primer	CGGTTTTACGAAGTAGGC
Reverse primer	CCCGATACCGCTACTATTAC
**POLR3B**: Forward primer	TTGATAGTTGGGGTTTAGG
Reverse primer	CGCACTTCACTAAACAACTC
**GIGYF2**: Forward primer	TTAGGATGGTAATTGCGAAG
Reverse primer	AAACCGACCTAACACTACCC

### RNA extraction

The trizol reagent was utilized to extract total RNA from both normal and tumor samples. Liquid nitrogen was used to grind 100–200 mg of tissue. These crushed samples were mixed with 1 ml of trizol. After that, 400 µl of chloroform was added and was allowed to incubate for 15 min room temperature followed by centrifugation at 12,000 rpm at 4^o^C. Isopropanol was added in equal amounts to the supernatant and tubes were incubated for 15 min at room temperature. Further samples were centrifuged at 12,000 rpm at 4^o^C and pellets were washed using 80% ethanol. The pellet was air dried, followed by the addition of 25 µl of DEPC water and was stored at -80 °C. The purity and the concentration of the sample were measured using a micro-volume spectrophotometer (DeNovix DS-11).

### Expression analysis through quantitative real-time RT-PCR

We isolated total RNA from normal (*n* = 16) and ovarian cancer tissues (*n* = 46). Table 2 displays the details of the samples used. 1 µg of total RNA was reverse transcribed using the QuantiTect reverse transcription kit (Qiagen). To analyze DMG expression, a real-time PCR machine (Applied Biosystems StepOne Plus) was employed. cDNA samples were used as the template for quantitative PCR with SYBR green master mix (Thermo Fisher, Scientific), with a final volume of 10ul. For the analysis of the PCR data, Excel, Graph Pad Prism (8.0.1), and StepOne software v2.3 were used. RT-PCR primers (Table 3) were designed using the Primer Blast (NCBI) and subsequently validated using insilico PCR, and net primer software. To avoid replication errors, each reaction was carried out in triplicates. β-actin served as a reference gene. PCR cycling conditions are as follows; ten minutes at 95 °C, fifteen seconds at 95 °C (melting), and thirty seconds at 55 to 58 °C (annealing and extension). Target gene relative expression was determined by applying the Livak method (2-∆∆Ct). To ascertain statistical significance, we used one-way ANOVA, the mean, standard deviation, and the Student’s t-test with a two-tailed distribution. Statistical significance was determined using *p*-values of 0.05.

**Table 2:** Detalis of cDNA samples used for QRT-PCR expression analysis

Tissue (*n* = 62)		
Variables	Case	Control
**Age, n(%)**		
≤ 45	15(24.19%)	4(6.45%)
≥ 45	31(50%)	12(19.35%)
**Histological type, n(%)**		
Mucinous	7(15.21%)	**-**
Serous	30(65.21%)	**-**
Clear Cell	5(10.86%)	**-**
Endometroid	4(8.69%)	**-**
**FIGO stage, n(%)**		
I-II	19(41.30%)	**-**
III-IV	27(58.69%)	**-**
**Serum CA125 (U/ml)**		
	0.17 ± 980	12.3 ± 38.5

**Table 3:** Primer details of hypermethylated genes for expression analysis by QRT-PCR

Gene	Primer Sequence (5’-3’)	Length	Tm	GC%	Product Length
STK4	Forward- ACGGTACAGCTGAGGAACCC Reverse-GCTGCCATAGGACCCTTCTCC	20 21	61.54 62.26	60.00 61.90	123 bp
BMP2	Forward- GGGACCCGCTGTCTTCTAGC Reverse-CGCAACTCGAACTCGCTCAG	20 20	62.30 61.67	65.00 60.00	158 bp
CRKL	Forward- ACCCCGACTCACCTTGTGTG Reverse-GTGCAGAACTCAAGCTCGCC	20 20	61.33 62.41	60.00 60.00	164 bp
PLXND1	Forward- TGGGAAACTGATGGGGATCGT Reverse- AGCACGTAGGAGAAGCGGTC	21 20	61.18 61.94	52.38 60.00	117 bp
POLR3B	Forward-GCAGTTTGCTTGGTGCAGGG Reverse-CGTCCATGCTGCTCACGAAG	20 20	62.70 61.69	60.00 60.00	75 bp
GIGYF2	Forward- CTGGGTCAGCCTTTATGCCAAG Reverse-GCTAGTCGCCAACCTCCATC	22 20	61.53 60.53	54.55 60.00	158 bp

## Results

### MeDIP-seq analysis of genomic DNA

**Fig. 2:**
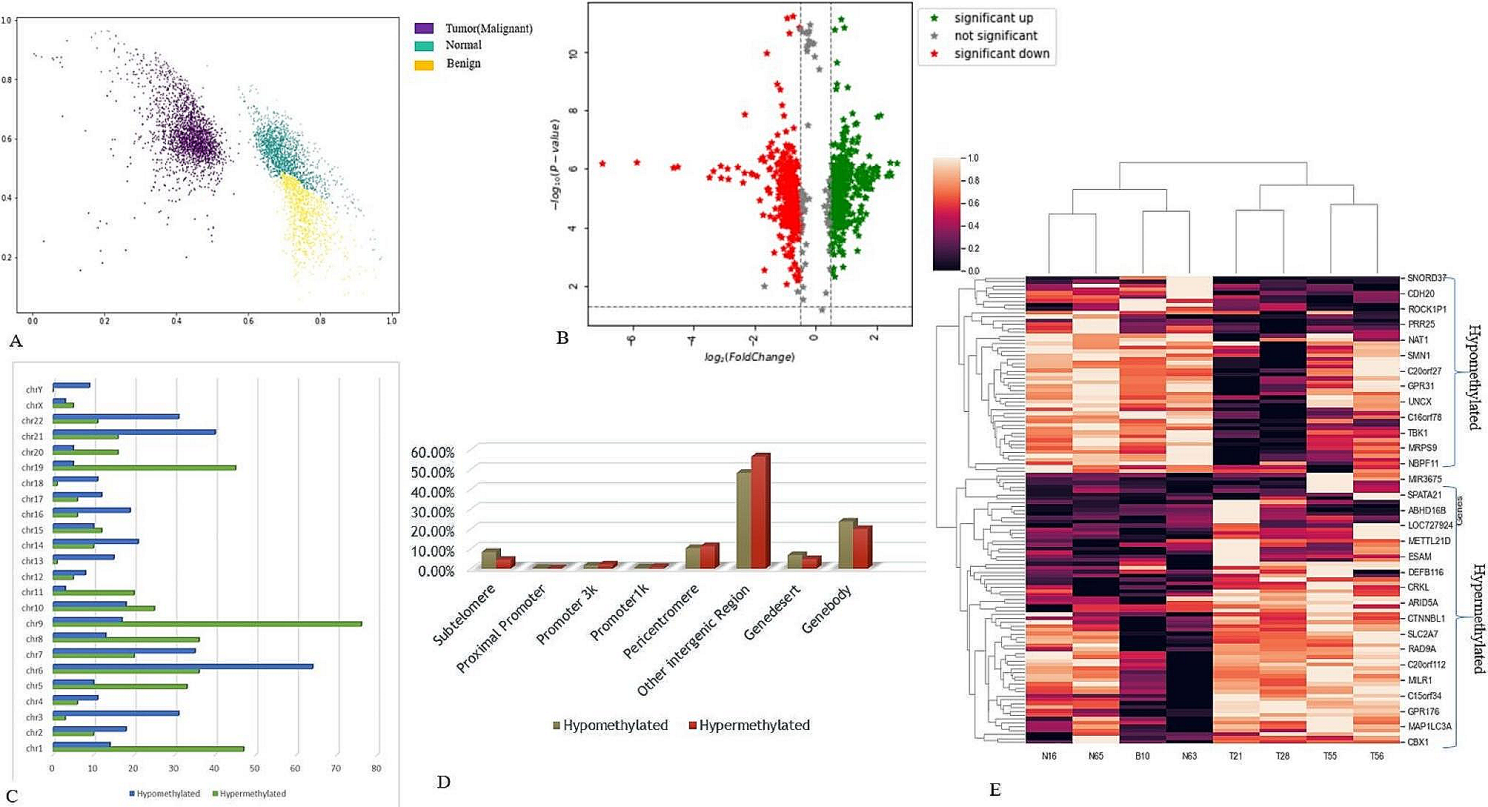
The genome-wide methylation profile of MeDIP-sequencing data of normal and ovarian cancer samples. (**A**) Principle component analysis of the genome-wide methylation profiles of Ovarian cancer and normal samples. (**B**) volcano plot shows the methylation profle of 2080 hypermethylated and 2194 hypomethy;ated DMRs (differentially methylated regions). (**C**) Chromosome wise distribution of hyper and hypomethylted DMRs. (**D**) Percentage of hyper and hypomethylated DMRs across different genomic regions. (**E**) Hierarchical clustering heatmap of common DMRs (68 hyper and 52 hypomethylated DMRs) of promoter region between normal and ovarian cancer samples

In this study, we performed the MeDIP-Seq analysis of the genomic DNA of ovarian cancer patients (*n* = 4), benign (*n* = 1), and healthy controls (*n* = 3). We observed the size distribution of g-DNA centered on 295 bp with a range of 212–418 bp. The MeDIP-Seq libraries were constructed with the genomic DNA derived from patients, and healthy controls were subjected to next-generation sequencing. The MeDIP-seq libraries were sequenced with Illumina NextSeq 500. After sequencing, low-quality sequences and adapter sequences were removed. Consequently, on average, 63 million and 72 million raw sequenced reads were acquired for patients and controls respectively, of which 79.02% and 89.8% were aligned to the reference genome (Human hg37) (Table 4). Under accession number GSE244405, the raw MeDIP-Seq data is available to the NCBI database. We observed the distinct methylation pattern revealed through principal component analysis (Fig. 2A). The volcano plots depicted the distribution of DMGs (Fig. 2B).

**Table 4:** MeDIP-seq statistics summery of ovarian cancer and normal samples

Sample	Total number of Reads	Number of mapped reads	Mapped reads rate	Number of bases
**T1**	6,24,76,582	11,62,17,593	93.01%	9,37,77,27,760
**T2**	7,31,78,135	13,60,78,453	92.98%	10,98,61,26,578
**T3**	5,66,04,730	3,25,19,194	57.44%	8,35,14,58,223
**T4**	6,15,20,612	4,39,16,362	71.38%	9,08,70,06,821
**N1**	7,23,08,042	13,32,98,461	92.17%	10,85,75,37,884
**N2**	8,00,52,434	14,83,76,154	92.67%	12,01,84,82,958
**N3**	6,37,61,008	5,39,17,982	84.56%	8,97,38,86,283
**B1**	6,22,02,086	4,99,57,304	80.31%	8,69,94,85,398

### Differentially methylated regions in ovarian cancer samples

Genome-wide DNA methylation data analysis of ovarian cancer and healthy patients ended with 4274 DMRs by applying criteria *p*-value < 0.05 and fold change > 0.1. Out of which, 2080 were hypomethylated, and 2194 were hypermethylated, indicating the presence of more hypermethylated fragments. The DMRs were found to be located in promoter 1k (0.7%), promoter 3k (1.82%) and proximal promoter (0.23%), Gene body (21.78%), Gene desert (5.76%) intergenic regions (52.5%), pericentromeric region (10.83%), and subtelomeric regions (6.36%) (Fig. 2D). Chromosome-wise distribution of DMRs is shown in Fig. 2C. Most of the DMRs were concentrated in the gene body and intergenic regions and a small fraction were in the promoter region. We obtained 120 DMRs present in the promoter regions, including 68 hypermethylated and 52 hypomethylated DMRs. We further sorted the top 40 hypermethylated DMRs based on *p*-value < 0.05 and fold change > 0.3, literature survey, and the number of hypermethylated fragments. Some of the reported hypermethylated genes in ovarian cancer are BRCA1, RASSF1A, TGFBI, DOK1, RUNX3, and CAMK2N1 [25–27]. The significant differential methylation profile of the top 40 genes is shown through a heatmap (Fig. 2E).

### Gene ontology and pathway analysis of DMGs

**Fig. 3:**
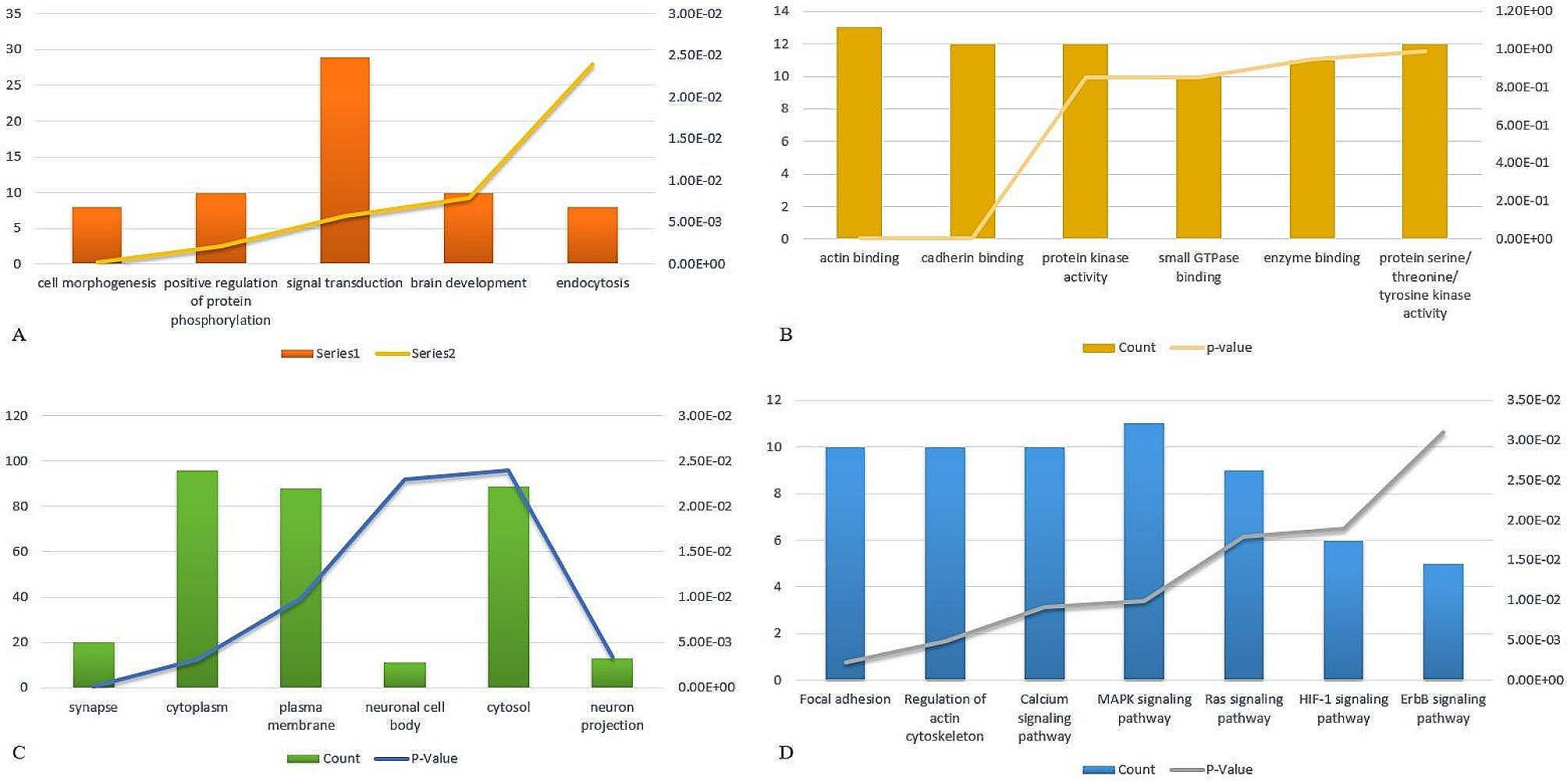
Functional investigation of differenetially methylated genes using DAVID tool (**A**) Biological function (**B**) Molecular function (**C**) Cellular component and (**D**) KEGG pathway

We conducted a GO (gene ontology) analysis of DMGs that was performed via the DAVID functional annotation tool to investigate the biological significance of hypermethylated genes. The result from the GO analysis includes three functional groups –Biological Process, Molecular Function, and Cellular components. In the biological process category, results indicated that most of the DMGs were associated with cell morphogenesis, signal transduction, and positive regulation of protein phosphorylation (Fig. 3A). Under molecular function analysis, DMGs were mainly involved in actin binding, cadherin binding, protein kinase activity, and protein serine/threonine kinase activity (Fig. 3B). Moreover, in cellular component analysis, DMGs were predominantly involved in the plasma membrane, cytoplasm, cytosol, and synapse (Fig. 3C). Furthermore, in the category of KEGG pathway analysis, DMGs are significantly enriched in focal adhesion, regulation of actin cytoskeleton, calcium signalling pathway, MAPK, and Ras signaling pathway (Fig. 3D). The results obtained from GO and KEGG pathway analysis are shown in Fig. 3 and detailed information in Table 5.

**Table 5:** Gene ontology and pathway analysis of hypermethylated genes using DAVID tool. 6 A) KEGG pathway, 6B) Biological process (BP), 6C) Cellular component (CC) and 6D) Molecular function (MF)

Category	Term	Count	*P*-Value
KEGG_PATHWAY	Focal adhesion	10	2.20E-03
	Regulation of actin cytoskeleton	10	4.90E-03
	Calcium signaling pathway	10	9.20E-03
	MAPK signaling pathway	11	9.90E-03
	Ras signaling pathway	9	1.80E-02
	HIF-1 signaling pathway	6	1.90E-02
	ErbB signaling pathway	5	3.10E-02
GOTERM_BP_DIRECT	cell morphogenesis	8	2.40E-04
	positive regulation of protein phosphorylation	10	2.10E-03
	protein autophosphorylation	9	2.60E-03
	synapse assembly	6	2.70E-03
	eye development	5	2.80E-03
	modulation of synaptic transmission	6	5.00E-03
	protein targeting to lysosome	4	5.80E-03
	signal transduction	29	5.80E-03
	response to thyroid hormone	3	5.80E-03
	brain development	10	7.90E-03
	homophilic cell adhesion via plasma membrane adhesion molecules	8	8.30E-03
	establishment of cell polarity	4	1.20E-02
	peptidyl-tyrosine phosphorylation	5	1.40E-02
	central nervous system development	7	1.40E-02
	detection of calcium ion	3	1.40E-02
	animal organ development	4	1.40E-02
	positive regulation of epithelial cell proliferation	5	1.50E-02
	response to nicotine	4	1.50E-02
	actin filament organization	7	1.60E-02
	endocytosis	8	2.40E-02
	positive regulation of bone resorption	3	2.50E-02
	epidermis development	5	2.60E-02
	neuromuscular synaptic transmission	3	3.10E-02
	transmembrane receptor protein tyrosine kinase signaling pathway	6	3.30E-02
	negative regulation of cell migration	7	3.60E-02
	animal organ morphogenesis	6	3.60E-02
	glial cell differentiation	3	3.90E-02
	fibroblast growth factor receptor signaling pathway	4	4.10E-02
	multicellular organism development	6	4.20E-02
	regulation of cytoskeleton organization	3	4.20E-02
	positive regulation of osteoclast differentiation	3	4.20E-02
	cell-cell adhesion	7	4.40E-02
	regulation of cell shape	6	4.80E-02
	protein dephosphorylation	6	4.90E-02
GOTERM_CC_DIRECT	synapse	20	8.80E-05
	cytoplasm	96	3.10E-03
	neuron projection	13	3.40E-03
	axon terminus	5	7.30E-03
	axon	12	7.90E-03
	adherens junction	8	9.80E-03
	plasma membrane	88	9.90E-03
	sarcolemma	6	1.30E-02
	neuronal cell body	11	2.30E-02
	cytosol	89	2.40E-02
	presynaptic membrane	6	2.60E-02
	integral component of postsynaptic density membrane	4	2.90E-02
	postsynaptic density membrane	5	3.80E-02
	extrinsic component of postsynaptic membrane	2	3.90E-02
	cell-cell junction	7	4.00E-02
	cell projection	7	4.00E-02
	glutamatergic synapse	11	4.40E-02
GOTERM_MF_DIRECT	actin binding	13	2.50E-03
	cadherin binding	12	4.20E-03
	protein phosphatase binding	6	7.60E-03
	heterocyclic compound binding	3	7.70E-03
	alpha-catenin binding	3	9.30E-03
	dystroglycan binding	3	9.30E-03
	protein kinase activity	12	1.50E-02
	SH2 domain binding	4	1.80E-02
	small GTPase binding	10	1.90E-02
	protein kinase activator activity	4	1.90E-02
	clathrin binding	4	2.30E-02
	calmodulin binding	8	2.30E-02
	phospholipid scramblase activity	3	2.40E-02
	phosphatidylinositol-3-phosphate binding	4	2.40E-02
	SH3 domain binding	6	2.90E-02
	3’,5’-cyclic-nucleotide phosphodiesterase activity	3	3.20E-02
	protein tyrosine kinase binding	4	3.30E-02
	enzyme binding	11	3.50E-02
	protein serine/threonine/tyrosine kinase activity	12	3.90E-02
	structural molecule activity	7	4.90E-02

### PPI network of hub genes

**Fig. 4:**
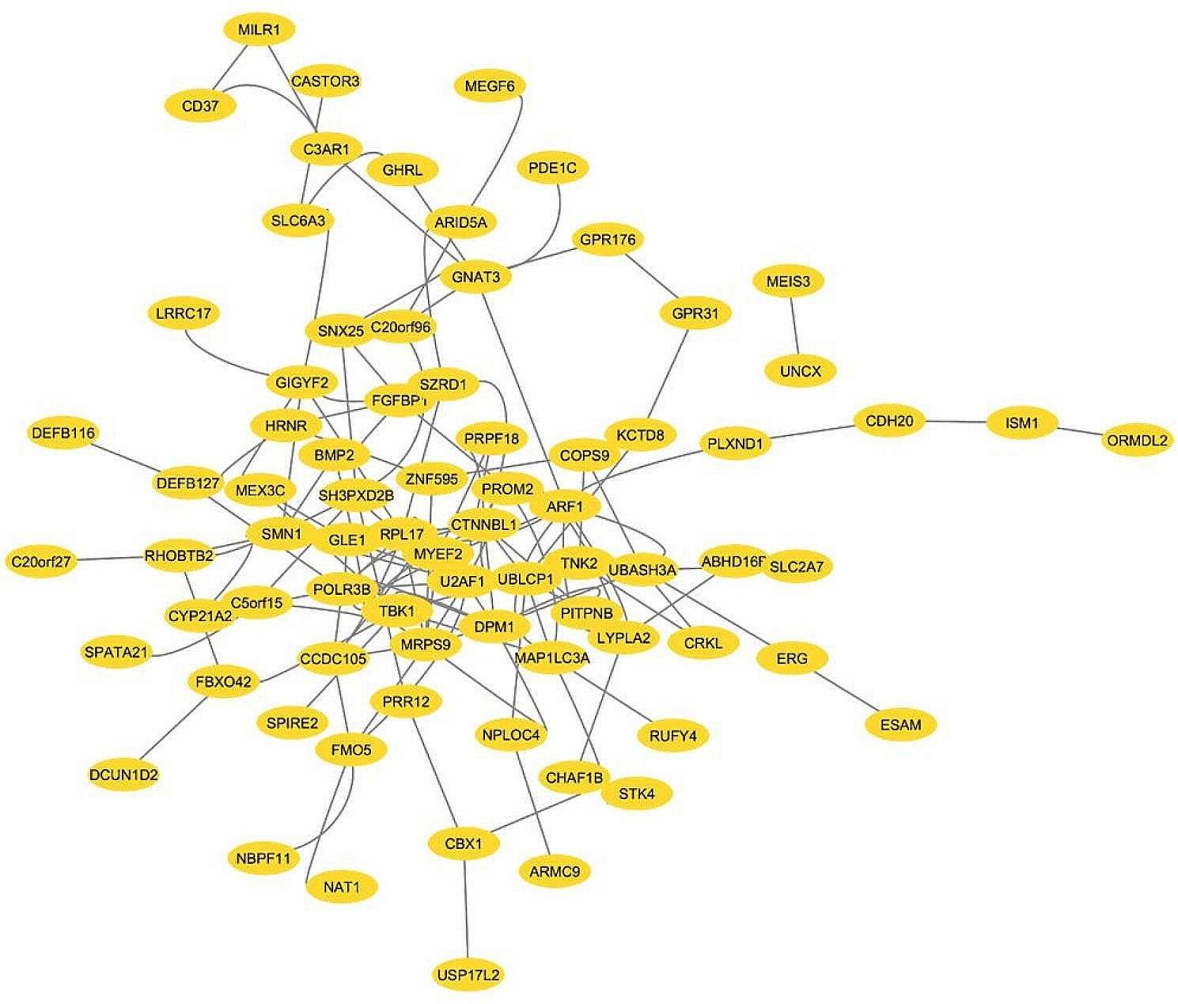
Protein-Protein interaction network of DMRs of promoter region analysed using STRING online database and cytoscape tool

To further explore the interaction between DMGs, a protein network comprising 118 DMGs was created using the STRING database and Cytoscape. In this network, isolated nodes have been removed, resulting in a fully formed network containing 86 nodes and 134 edges (Fig. 4). The generated network was imported into Cytoscape and analyzed by a network analyzer, resulting in distinguishable nodes based on degree value. The top 10 nodes were filtered based on degree value and betweenness centrality, and nodes with higher values (degree ≥ 10) were subsequently considered as hub nodes [28]. The top 6 hub nodes were CRKL, BMP2, POLR3B, PLXND1, STK4 and GIGYF2. Among them, POLR3B and GIGYF2 are novel targets and were not reported previously in OC. The details of these hypermethylated genes are shown in Table 6.

### Validation by targeted bisulfite sequencing

**Fig. 5:**
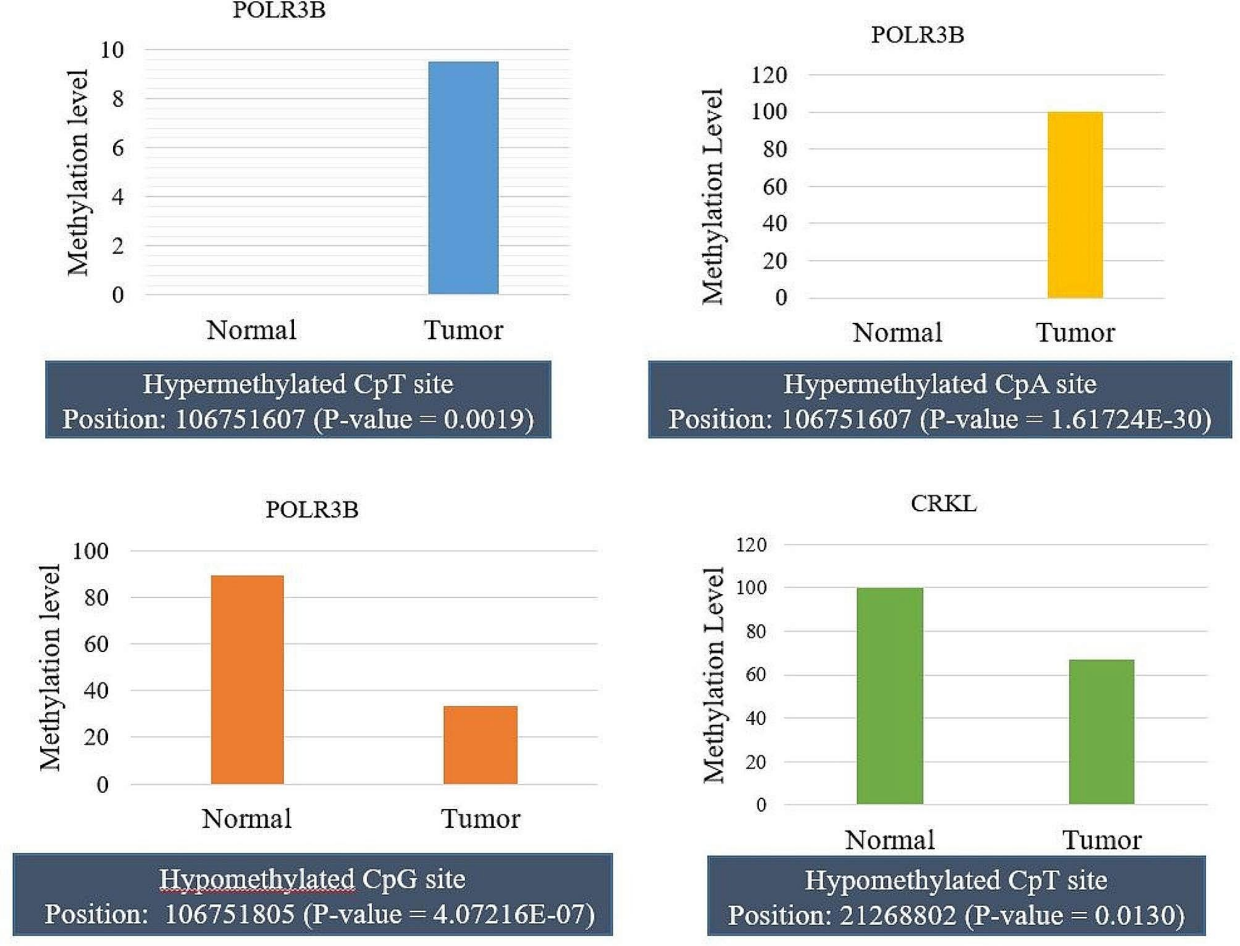
The methylation level of CpG and non-CpG sites of POLR3B and CRKL between tumor and normal samples obtained from targeted sequencing

We performed the targeted bisulfite sequencing to check the consistency of region-specific methylation among CpG sites in all six hypermethylated genes obtained from MeDIP-Seq data. Gene location obtained from MeDIP-Seq data was analyzed through NCBI. Depending upon the number of CpG sites and maximum length (1000 bp), regions of promoters were selected for targeted sequencing (Table 7). Targeted sequencing data analysis demonstrated a significant difference in the methylation status of 3 sites of the POLR3B DMR region (900 bp). Of them, two non-CpG sites [CpT-106,751,607 (*p*-value = 0.0019); CpA-106,751,607 (*p*-value = 1.61724E-30)] were significantly hypermethylated and one CpG site (CpG-106,751,805) was significantly hypomethylated (*P*-value = 4.07216E-07). In CRKL, one non-CpG site (CpT-21,268,802 (*p*-value = 0.0130)) was significantly hypomethylated. The results are shown in Fig. 5; Table 8. We observed significant CpG methylation along with non-CpG methylation (CpA and CpT). According to the literature, CpA accounts for 25% of methylation of all methylated cytosines and is one of the most frequent one after CpG, followed by CpT [29].

**Table 6:** Details of top six hypermethylated genes obtained from MeDIP-sequencing

Hypermethylated Genes	Gene name	Feature	log2FoldChange	*p*-value	Status in ovarian cancer
POLR3B	RNA polymerase III subunit B	Promoter 1k	0.66	0.006106	Not reported in ovarian cancer
PLXND1	Plexin D	Promoter 1k	0.69	0.000595	Only 1 study reported mentioning regulation of Epithelial-Mesenchymal transition by PLXND1
GIGYF2	Grb 10-interacting GYF protein 2	Promoter 1k	0.33	0.033441	Not reported in ovarian cancer
STK4	Serine/Threonine kinase 4	Promoter 3k	0.5	0.019861	Reported as downregulated in OC
CRKL	CRK-like proto oncogene	Promoter 3k	0.78	0.003422	Two studies were reported demonstrating the regulation of EMT through ERK signalling pathwway. Another study reported its overexpression.
BMP2	Bone morphogenetic protein 2	Promoter 3k	0.5	0.023133	Few studies were reported. One of them had shown a downregulated expression.

**Table 7:** Information of differentially methylated gene fragment used for targeted sequencing

Gene Name	Total Number of CpG	Total length	Region
STK4	11	650 bp	chr20:43,597,001–43,597,650
BMP2	6	750 bp	chrX:6,750,901-6,751,650
CRKL	14	1000 bp	chr22:21,268,451 − 21,269,450
PLXND1	127	1000 bp	chr3:129,324,301 − 129,325,300
POLR3B	65	900 bp	chr12:106,751,401 − 106,752,300
GIGYF2	69	850 bp	chr2:233,561,551 − 233,562,400

### Expression analysis of DMGs

We conducted RNA sequencing of 4 tumors and 4 normal samples (Accession No. GSE244405). Only the POLR3B gene showed significant downregulation in RNA-Seq data. Further, we looked for the expression pattern of these hypermethylated genes in GEO expression datasets (GSE54388, GSE38666, GSE4122). The results revealed significant downregulation of all genes STK4, GIGYF2, POLR3B, BMP2, and CRKL except PLXND1 (Table 9).

**Table 8:** Results of targeted sequencing showing methylation details of non-CpG sites of POLR3B and CRKL hypermethylated gene

Gene	Gene start	Gene end	Methylation Position	Control Methylation %	Treatment Methylation %	*p*Value
POLR3B	106,751,601	106,751,610	106,751,607	0	9.523809524	0.001953564
POLR3B	106,751,701	106,751,710	106,751,705	0	99.89007179	1.61724E-30
POLR3B	106,751,801	106,751,810	106,751,805	89.33610575	33.33333333	4.07216E-07
CRKL	21,268,801	21,268,810	21,268,802	100	66.66666667	0.013078531

**Table 9:** Expression values of hypermethylated genes obtained from GEO expression datasets, RNA-seq data (GSE212991) and validated values from QRT-PCR

Hypermethylated Gene	Expression Values (*p*-Value)			
	GSE54388	GSE38666	RNA-seq	QRT-PCR
POLR3B	0.382	0.00598	0.0006585	0.0011
CRKL	0.624	0.0113	--	0.0401
STK4	0.000791	0.75	--	0.0008
GIGYF2	0.0175	0.68	--	0.0097
BMP2	1.62E-07	8.11E-09	--	0.0003
PLXND1	0.557	--	--	0.0925

**Fig. 6:**
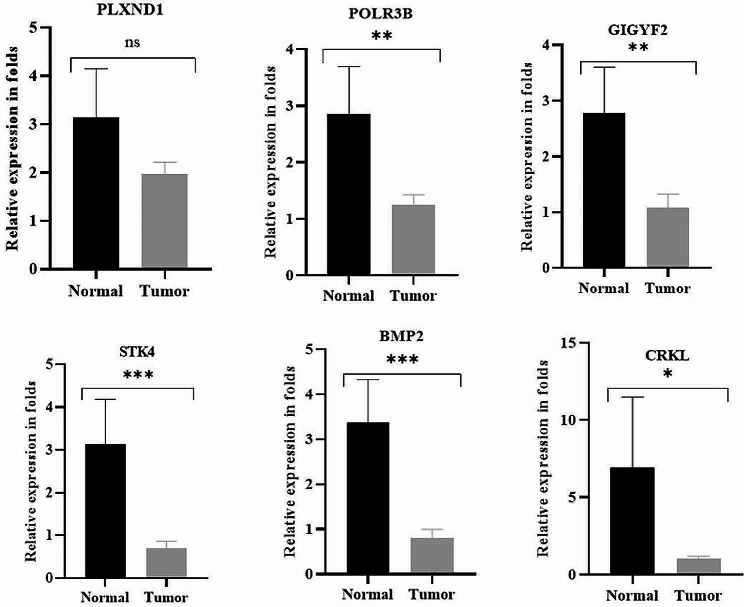
The relative mRNA expression of top six hypermethylated genes using QRT-PCR between normal (*n* = 16) and ovarian cancer (*n* = 46) samples. The error bars associated with the data represent the standard error of the mean, and the height of each box represents the mean value of sample-specific fold change (2^−△△ct^) values. To calculate *p*-values, Student’s t-test was employed. *p < 0.05, **p < 0.01, ***p < 0.001, ns- non significant

We also performed validation through quantitative RT-PCR analysis and observed that all hypermethylated genes [(POLR3B (*p* = 0.0011), STK4 (*p* = 0.0008), BMP2 (*p* = 0.0003), GIGYF2 (*p* = 0.0097), CRKL (*p* = 0.0401)] exhibited significant downregulation except PLXND1. The results are shown in Fig. 6.

## Discussion

Regardless of significant progress in surgical and medical therapy, ovarian cancer still holds the highest mortality rate among all other gynecologic malignancies. Challenges persist in detecting ovarian cancer at an early stage as there are no distinct clinical signs, no accurate and efficient early detection biomarkers, and no potent treatment strategies for advanced-stage patients. Therefore, it is vital to comprehend the molecular mechanisms behind the tumor progression, which could be further investigated to improve the overall survival rate of ovarian cancer patients and thereby prevent disease recurrence.

Methylation plays an essential role in tumorigenesis, and aberrant methylation is thought to be the most frequent initial molecular change in carcinogenesis. Therefore, the examination of site-specific methylation profiles has a great potential to narrow down a panel of epigenetic biomarkers for early diagnosis of ovarian cancer [30, 31]. Moreover, many studies have also reported the association of promoter hypermethylation with gene silencing, thereby making it a significant aspect of biomarker-related studies. Costello et al. first reported the existence of a specific hypermethylation pattern in CpG islands in many types of malignancies, including ovarian cancer, and was later verified by Esteller et al. [28].

In this study, we used MeDIP-Seq, a cost-effective high-throughput method, to investigate the differential methylation pattern in epithelial ovarian cancer to explore the candidate DMGs and potential pathways regulated by them which on further validation could act as effective potential targets for ovarian cancer detection and treatment. Through MeDIP-Seq analysis, 4,768 DMRs were listed comprising a higher proportion of hypermethylated DMRs than hypomethylated ones. Furthermore, a significant proportion of hypermethylation DMRs was found in the intergenic region and gene body, while a small percentage was present in the promoter region. The top 40 hypermethylated DMRs, existent in the promoter region were considered for further analysis. Functional analysis revealed that these DMGs were strongly related to various biological processes, such as positive regulation of protein phosphorylation, signal transduction, actin binding, and protein serine/threonine/tyrosine kinase activity. Pathway enrichment analysis revealed that most DMGs were mainly associated with the MAPK signaling pathway, regulation of actin cytoskeleton, calcium signaling pathway, focal adhesion, and Ras signaling pathway. The majority of them have a tight relationship with the onset and development of ovarian cancer.

As reported by previous studies, focal adhesions are essential because they act as a bridge between the internal structure (actin cytoskeleton) and the extracellular matrix of the cell. This connection is crucial for motility, differentiation, survival, and cytoplasmic signalling of the cell [32]. MAPK pathway also plays a crucial role in the development of ovarian cancer as it is integrated into many cellular processes like apoptosis, cell growth, and proliferation which are the critical hallmarks of cancer development [33]. Low-grade serous ovarian carcinoma (LGSOC) often contains active MAPK mutations [34]. In human malignancies, including ovarian cancer, Ras is one of the most frequently altered signaling pathways. Ras is involved in several other pathways that control cell migration, cell adhesion, survival, cell growth, and differentiation. Among ovarian cancers, KRAS mutations are commonly detected as one of the most frequent abnormalities [35]. Therefore, it is interesting to study these pathways as they are associated in numerous processes of cell development and growth.

Protein-protein network analysis is essential to analyze the interactions between DMGs for molecular evaluation of numerous diseases. Therefore, to interpret the centrality role of DMGs, the PPI network of the top 40 DMGs was constructed. Further top 6 hypermethylated hub genes, namely POLR3B, PLXND1, GIGYF2, STK4, CRKL, and BMP2 were identified in this study.

Validation of the methylation level of DMRs at individual CpGs of the promoter region through targeted bisulfite sequencing on the NGS platform was carried out. Cytosine methylation in CpG is essential for cellular development and proliferation. However, non-CpG (CpA, CpT, and CpC) methylation is also present in different cell types and stages of cell development. Most of the non-CpG methylation is found in stem cells and pluripotent cells. The function of non-CpG methylation is still unclear however it is associated with altered gene expression. CpA methylation is reported to be the highest (about 12%) followed by CpT and CpC [36]. A few studies have demonstrated the functional association of non-CpG methylation in breast cancer, brain lymphoma, and prostate cancer [37]. Some cancer-related genes also showed methylation at non-CpG sites like NOTCH3, GSTP1, and TP53 [38–40]. In primary effusion lymphoma, the B-cell-specific B29 gene gets silenced due to non-CpG promoter methylation. Methylation was reported in densely clustered non-CpG regions in lung carcinoma [41, 42]. Studies highlighting the non-CpG methylation in cancer underscore the importance of investigating and comprehending the functional role of site-specific non-CpG methylation in the context of cancer epigenetics.

Targeted bisulfite sequencing analysis of POLR3B and CRKL gene revealed significant non-CpG methylation at particular loci. This study attempts to contrast non-CpG methylation at CpG islands between ovarian cancer and normal ovarian tissues.

In this study, two novel hypermethylated genes (POLR3B and GIGYF2) are reported with their expression levels in EOC. Limited reports are available related to expression levels of the other four hub genes (STK4, BMP2, CRKL, and PLXND1). POLR3B (RNA polymerase III subunit B) is the largest subunit of RNA pol III and participates in the transcription of rRNA and tRNA genes. Luo et al. identified a biomarker panel having POLR3B, which could significantly differentiate the stage I tumor patients in lung adenocarcinoma [43]. Similarly, Han et al. discovered 9 gene panel biomarkers containing POLR3B as a prognostic indicator of bladder cancer [44]. Targeted bisulfite sequencing results confirmed a significant difference in the methylation status of 3 sites of the POLR3B DMR region (a CpG island of 900 bp). Two non-CpG sites [CpT and CpA] were significantly hypermethylated and one CpG site was significantly hypomethylated. POLR3B hypermethylation was also correlated with its expression in our study.

GIGYF2 (Grb 10- interacting GYF protein 2) is known to regulate multiple signaling pathways involved in neural development. Zhu et al. reported the downregulation of GIGYF2 resulting in suppression of gastric cancer and gliomas [45, 46]. Promoter methylation of GIGYF2 was correlated with its downregulation in the present study.

PLXND1 (Plexin D1) is a receptor for semaphorin, SEMA3E, which is crucial in regulating migration and cell proliferation and scores a strong therapeutic potential [47]. Li et al. reported the prognostic significance of PLXND1 in hepatocellular carcinoma [48]. PLXNDI, in conjugation with SEMA3E, enhances the Epithelial-Mesenchymal Transition by activating the PI3/Akt signaling pathway in colorectal cancer and through SEMA3E in endometrioid cancer [49, 50]. Association of PLXND1 with angiogenesis and cell migration is reported in cervical and prostate cancer respectively [51, 52].

CRKL (CRK-like proto-oncogene) is a cell signaling protein with one SH2 domain and two SH3 domains, facilitating interactions between proteins. Overexpression of CRKL may promote proliferation and invasion through the ERK signaling pathway in pancreatic cancer, breast cancer, small-cell lung cancer, gastric cancer, and myeloma [53–57]. CrkL is reported to control the CCL19/CCR7-induced EMT through the ERK signaling pathway in EOC [58, 59]. Our results indicate CRKL as a hypermethylated gene but on validation through targeted sequencing, one hypomethylated non-CpG site (CpT-21,268,802 (*p*-value = 0.0130) turned out to be very significant in discriminating ovarian cancer from non-cancerous state.

BMP2 (Bone morphogenetic protein 2) is a member of TGF-β (tumor growth factor-β). BMP2 is the most extensively investigated protein contributing to the development of bones, EMT, and multiple signaling pathways [60]. High BMP2 expression is a promising therapeutic target in lung cancer [61]. In contrast, the downregulation of BMP2 in colorectal cancer further impedes DNA replication and chemotherapy resistance [62]. BMP2 causes the invasion and proliferation of gastric cancer cells through the activated PI3/Akt signalling pathway [63, 64]. Contrasting reports on the correlation between overexpressed BMP2 and poor prognosis and reduced BMP2 expression with a worse prognosis have been documented [65, 66]. Fukuda et al. observed elevated BMP2 expression in ovarian cancer patients after chemotherapy [67].

STK4 (Serine/Threonine Kinase 4) is a key member of the hippo signaling pathway. It is also engaged in the AKT signaling pathway. Ready et al. reported that STK4 inhibits cancer cell proliferation by regulating crucial oncogenic pathways, encompassing DNA repair and cell cycle regulation [68–72]. Peng et al. showed that STK4 methylation mediated downregulation consequently facilitate the progression of thyroid carcinoma by activating the Hippo signaling pathway [73]. Promoter hypermethylation of STK4 was also reported in in human sarcomas and pancreatic cancer [74, 75].

Our study presents hypermethylated CGI of POLR3B, GIGYF2, and PLXND1 with the H3k27 mark. Our preliminary results highlight targets (PLXND1, POLR3B, CRKL, GIGYF2, BMP2, and STK4) showing a negative correlation between promoter methylation gene expression. We also reported significant downregulation of these hypermethylated genes when analyzed through two GEO datasets (GSE5388 and GSE38666) and RNA-seq data (GSE212991).

## Conclusion

This investigation showcased a comprehensive genome-wide methylation profile of epithelial ovarian cancer (EOC) and identified six hypermethylated/downregulated genes, (POLR3B, PLXND1, GIGYF2, CRKL, STK4, and BMP2) as potential diagnostic targets. The study also emphasized the potential of non-CpG sites to discriminate ovarian cancer from the disease-free normal sample. Network analysis highlighted the pathways crucial to cancer development, including the focal adhesion, Ras signaling pathway, and MAPK signaling pathway. POLR3B and GIGYF2, identified as novel hypermethylated genes, might serve as promising biomarkers for diagnosing and predicting the prognosis of ovarian cancer. These newly identified hypermethylated/downregulated genes warrant further investigations regarding their potential as therapeutic targets.

### Electronic supplementary material

Below is the link to the electronic supplementary material.

Supplementary Material 1

## Data Availability

The Medip-seq data used in this investigation is available in the Gene Expression Omnibus (GEO) database (https://www.ncbi.nlm.nih.gov/geo/) with accession number GSE244405. Expression datasets were GSE212991, GSE54388, and GSE38666.
