# Supplementary material for: Comprehensive DNA methylation profiling by MeDIP-NGS identifies potential genes and pathways for epithelial ovarian cancer
Source: J Ovarian Res. 2024 Apr 16;17:83. doi: 10.1186/s13048-024-01395-3 (PMC11022481; doi:10.1186/s13048-024-01395-3)
Supplement: Supplementary file 1 — Supplementary Material 1 [file 13048_2024_1395_MOESM1_ESM.docx]

**Supplementary table 1.** Cilinal information of epithelial ovarian cancer patients

| **Sample name** | **Age(Range)** | **Stage** | **Histology** |
| --- | --- | --- | --- |
| T1 –T11 | 35-60 | I | Benign, clear cell carcinoma |
| T12-T20 | 28-57 | II | Serous |
| T21-T45 | 25-72 | III | Serous |
| T46-T61 | 37-60 | IV | Serous Adenocarcinoma |
| N1-N23 | 35-65 | Normal | Normal histology |
